# Supplementary material for: Gut Microbiota Associated with Clostridioides difficile Carriage in Three Clinical Groups (Inflammatory Bowel Disease, C. difficile Infection and Healthcare Workers) in Hospital Field
Source: Microorganisms. 2023 Oct 10;11(10):2527. doi: 10.3390/microorganisms11102527 (PMC10609531; doi:10.3390/microorganisms11102527)
Supplement: Supplementary file 1 [file microorganisms-11-02527-s001.zip › microorganisms-2622360-Supplementary-revised.pdf]

Supplementary materials

Table S1. Identification of patients and patients' information's CDI, IBD and HCW

Identification	Age	Sex	T1	T2	T3	ATB Before	Treatment for CDI
CDI patients							
**CDI 01**	38 y	F	X	X	NR	No	Vancomycin 5d
**CDI 02**	66 y	F	X	X	X	No	Vancomycin 24h
**CDI 03**	94 y	M	X	D	D	NG	NG
**CDI 04**	54 y	M	X	X	X	No	Vancomycin 6d
**CDI 05**	60 y	F	X	X	X	Cefuroxim and Ceftriaxon	No
**CDI 06**	59 y	F	X	X	X	No	Piperacilline and Tazobactam 7d
**CDI 07**	56 y	F	X	X	X	Cefuroxim	Vancomycin 10d
**CDI 08**	72 y	F	X	X	X	Moxiflocin	Vancomycin 10d
**CDI 09**	59 y	M	X	X	X	No	Vancomycin 7d
**CDI 10**	58 y	M	X	X	X	Ceftriaxon and norfloxacin	No
**CDI 11**	66 y	F	X	X	NR	Metronidazole	Amoxicillin and clavulanic acid
**CDI 12**	66 y	M	X	X	X	Ceftriaxon and Metronidazole	No
**CDI 13**	71 y	F	X	X	X	Sulfamethozole trimethoprim	Probiotic
**CDI 14**	55 y	M	X	X	X	No	Vancomycin
**CDI 15**	71 y	M	X	NR	X	No	Vancomycin 14d
IBD patients							
**IBD 01**	57	F	X	X	X	No	/
**IBD 02**	81 y	M	X	X	NR	No	/
**IBD 03**	26 y	F	X	NR	X	No	/
**IBD 04**	57 y	F	X	NR	X	No	/
**IBD 05**	24 y	F	X	X	X	No	/
**IBD 06**	49 y	F	X	X	X	ATB 1 month before the donation	/
**IBD 07**	38 y	F	X	NR	X	No	/
**IBD 08**	42 y	F	X	X	X	No	/
**IBD 09**	52 y	M	X	X	NR	No	/
**IBD 10**	60 y	F	X	X	X	No	/
**IBD 11**	30 y	F	X	NR	NR	No	/

**IBD 12**	61 y	F	X	NR	X	No	/
**IBD 13**	60 y	F	X	X	X	No	/
**IBD 14**	56 y	M	X	NR	NR	No	/
**IBD 15**	58 y	F	X	X	X	No	/
HCW patients							
**HCW 01**	42 y	M	X	X	X	No	/
**HCW 02**	25 y	M	X	X	X	No	/
**HCW 03**	56 y	F	X	X	X	No	/

Legend:

D: deceased; d: days; h: hours; y: years old, F: female; M: male; NR: not realized; NG: not given; red colour: positive sample.

Table S2. Classification of faeces with Bristol scale stool and means of preserving the samples

Samples	T1		T2		T3	
	Freezing	Scale	Freezing	Scale	Freezing	Scale
IBD 01	No	5	No	5	No	5
IBD 02	No	5	No	5	NR	NR
IBD 03	No	5	NR	NR	No	4
IBD 04	No	3	NR	NR	No	4
IBD 05	No	4	No	4	No	4
IBD 06	No	6	No	5	X	6
IBD 07	No	5	NR	NR	No	4
IBD 08	No	4	No	4	No	3
IBD 09	No	4	X	4	NR	NR
IBD 10	No	4	No	4	No	5
IBD 11	No	4	NR	NR	NR	NR
IBD 12	No	4	NR	NR	X	4
IBD 13	No	3	X	4	X	4
IBD 14	No	5	NR	NR	NR	NR
IBD 15	No	4	No	4	X	4
PS 01	No	3	No	3	No	3
PS 06	No	4	No	4	X	4
PS 11	No	4	No	4	X	4
CDI 01	No	4	No	5	NR	NR
CDI 02	X	6	X	6	No	4
CDI 03	No	4	NR	NR	NR	NR
CDI 04	X	7	No	7	X	5
CDI 05	X	3	X	5	No	4
CDI 06	X	5	No	4	X	4
CDI 07	X	6	X	5	No	4
CDI 08	X	6	X	5	No	4
CDI 09	X	5	No	5	X	4
CDI 10	X	6	X	4	X	5
CDI 11	X	7	X	7	NR	NR
CDI 12	X	5	X	5	X	3
CDI 13	X	5	No	5	X	4
CDI 14	X	6	X	6	X	4

CDI 15	X	7	NR	NR	X	4

Legend: X: Freezing; Score: 1 (constipation) to 7 (diarrhoea) (Bristol stool score) and 3 and 4 are normal faeces; NR: not realized; red colour: positive sample.

Table S3 – Significant taxa using linDA models in R studio v4.2.2 between *C. difficile* positive and *C. difficile* negative group.

	log_2FoldChange	*padj*
Clostridioides	3.03878103	1.38E-05
Colidextribacter	-1.8746591	0.0016972
Monoglobus	-1.9788814	0.0016972
UCG.003	-1.543897	0.00288813
Anaerostipes	-2.3853769	0.0032462
Enterococcus	3.2322434	0.00376198
Oscillibacter	-1.5685798	0.00376198
Clostridiales_ge	-1.6838486	0.00376198
Lachnospira	-2.3245454	0.00376198
Oscillospira	-1.1121663	0.00517723
Butyricicoccus	-1.7785305	0.00762989
Lachnospiraceae_NK4A136_group	-2.4230547	0.0085659
Clostridia_ge	-1.9161461	0.01312289
Enterobacterales_ge	1.77432568	0.01489544
Sutterella	-1.927567	0.01489544
Lachnospiraceae_UCG.004	-1.9382879	0.01489544
Romboutsia	-2.4449953	0.01489544
Oscillospiraceae_ge	-1.5896989	0.01716848
Fusicatenibacter	-2.6163993	0.01716848
Enterobacteriaceae_ge	2.18702628	0.02230039
Haemophilus	0.75252042	0.03028035
Coriobacteriales_ge	-1.3208606	0.03028035
Lachnospiraceae_ND3007_group	-2.0977945	0.03028035
Agathobacter	-2.2609146	0.03028035
Marvinbryantia	-1.1760316	0.03480656
Ruminococcaceae_ge	-1.7545244	0.03480656
Butyricicoccaceae_ge	-0.7260133	0.03513151
Rhodospirillales_fa_ge	-1.3629844	0.04454008
Bilophila	0.84579423	0.04778817

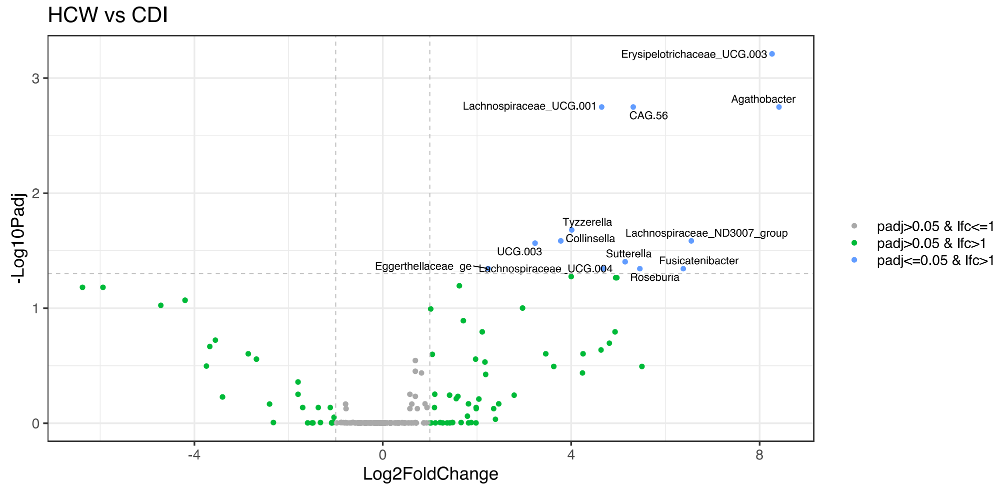

Figure S1. Group effect of bacterial microbiota. Linear model for differential abundance in Rstudio v4.2.2 (linDA) using groups and patients as variable. Here a representation of log_2fold change of the HCW group between CDI group. Two thresholds are present: The first one is fixed at $-log_{10}$(padj >0.05) and the seconde is fixed at -1 and +1 log_2fold change. The grey dots are the genera with *padj* > 0.05 and with an effect size ≤ 1; the green dots are the genera with *padj* > 0.05 and with an effect size > 1; the blue dots are the genera with *padj* ≤ 0.05 and with and effect size > 1.

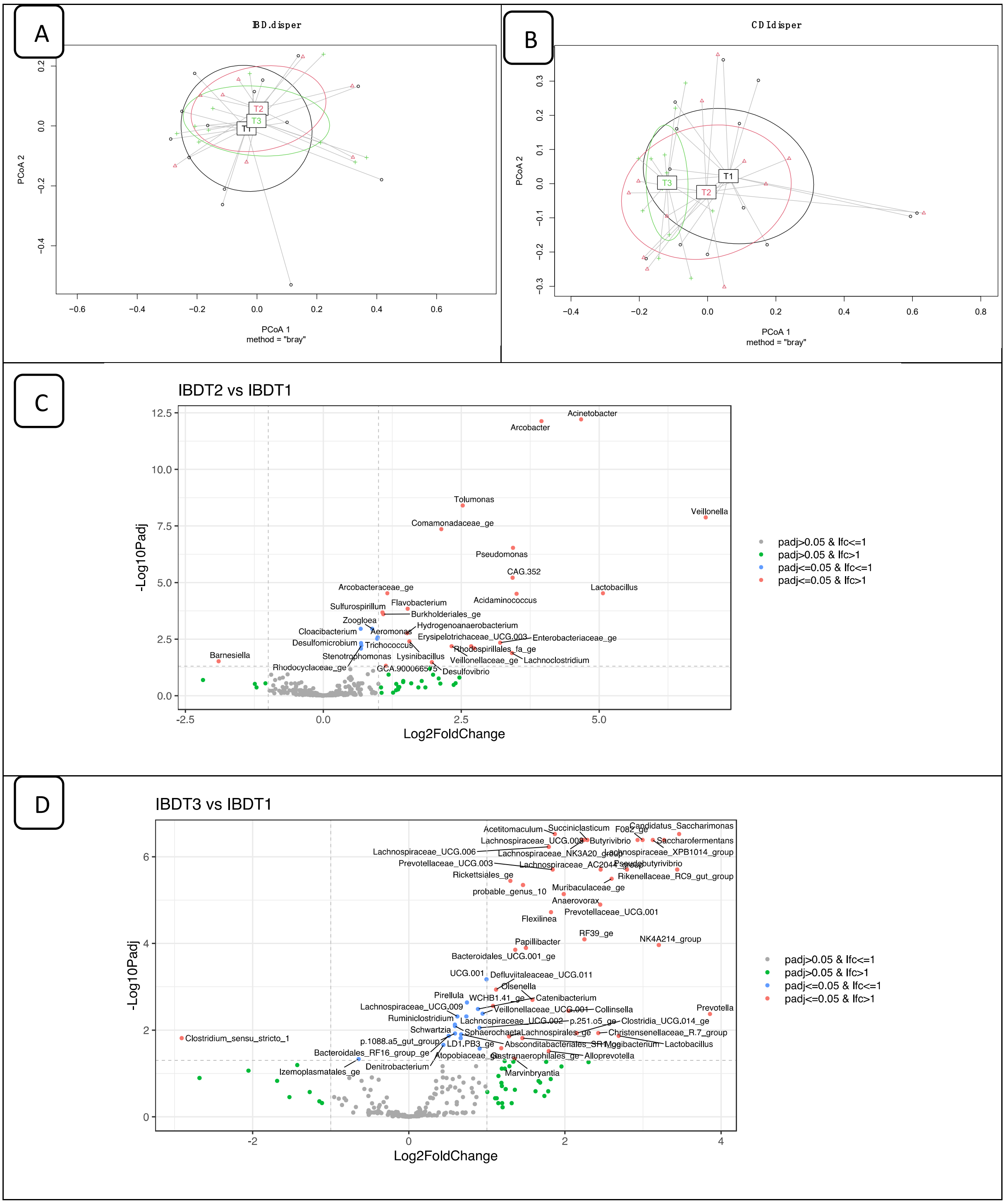

Figure S2 – Time effect on bacterial microbiota

Analysis of β-diversity using betadisper with vegan package in Rstudio v4.2.2. Betadisper analyzed beta-dispersion on the basis of Bray-Curtis dissimilarity matrix. Dissimilarity (beta dispersion) between groups was assessed using the Adonis$_2$ test. IBD time 1 group is represented in black, in IBD time 2 group is represented in red; in IBD time 3 group is represented in green;

Analysis of β-diversity using β-disper in Rstudio v4.2.2. CDI time 1 group is represented in black, in CDI time 2 group is represented in red; in CDI time 3 group is represented in green;

(C) Linear model for differential abundance in Rstudio v4.2. (linDA) using times and patients as variable for T2 vs T1 in IBD group. Here a representation of log_2fold change of the *C. difficile* positive group between *C. difficile* negative group. Two thresholds are present: the first one is fixed at -log_{10}(padj >0.05) and the second one is fixed at -1 and +1 log_2fold change. The grey dots are the genera with *padj* > 0.05 and with an effect size ≤ 1; the green dots are the genera with *padj* > 0.05 and with an effect size >1; the blue dots are the genera with *padj* ≤ 0.05 and with and effect size ≤ 1 and the red dots are the genera with *padj* ≤ 0.05 and with and effect size > 1.

(D) Linear model for differential abundance in Rstudio v4.2. (linDA) using times and patients as variable for T3 vs T1 in IBD group. Here a representation of log_2fold change of the *C. difficile* positive group between *C. difficile* negative group. Two thresholds are present: the first one is fixed at -log_{10}(padj >0.05) and the second one is fixed at -1 and +1 log_2fold change. The grey dots are the genera with *padj* > 0.05 and with an effect size ≤ 1; the green dots are the genera with *padj* > 0.05 and with an effect size >1; the blue dots are the genera with *padj* ≤ 0.05 and with and effect size ≤ 1 and the red dots are the genera with *padj* ≤ 0.05 and with and effect size > 1.

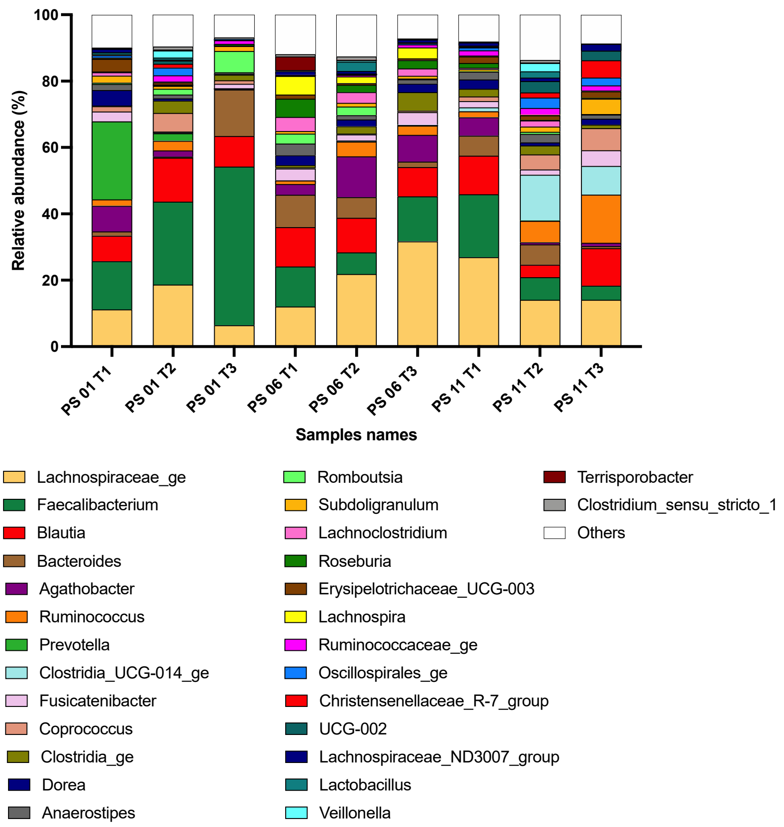

Figure S3 – Evolution of gut microbiota (mean cumulative relative abundance) of bacterial genera for clinical group through time in HCW groups

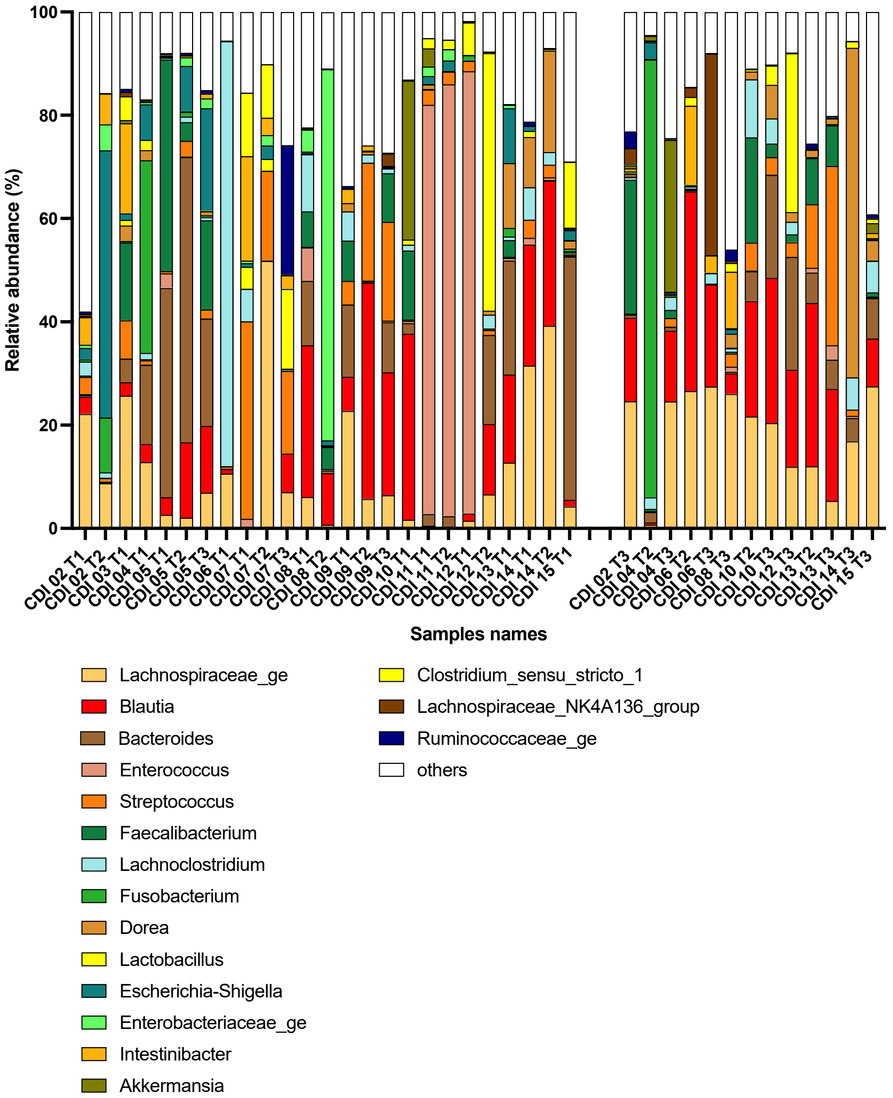

Figure S4 – Evolution of gut microbiota (mean cumulative relative abundance) of bacterial genera for clinical group through time in CDI groups

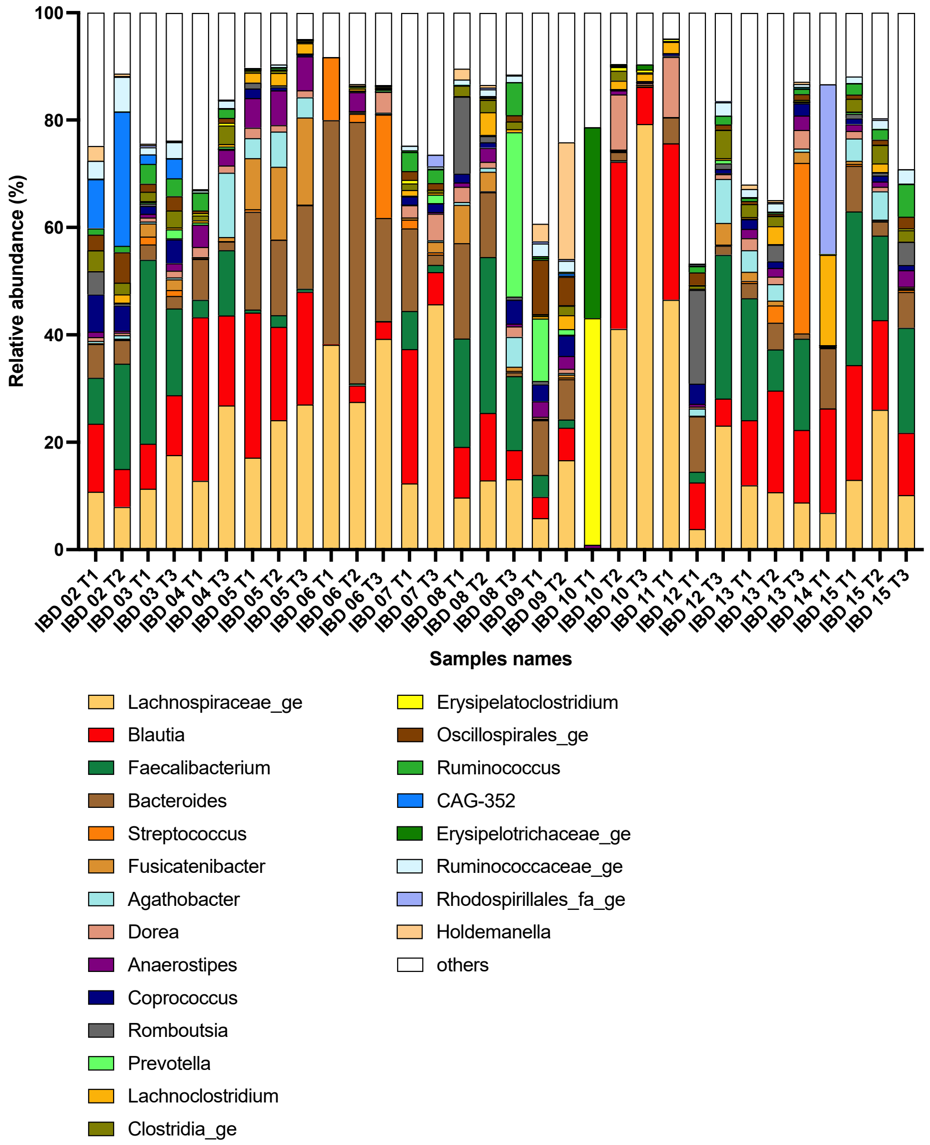

Figure S5 – Evolution of gut microbiota (mean cumulative relative abundance) of bacterial genera for clinical group through time in IBD groups

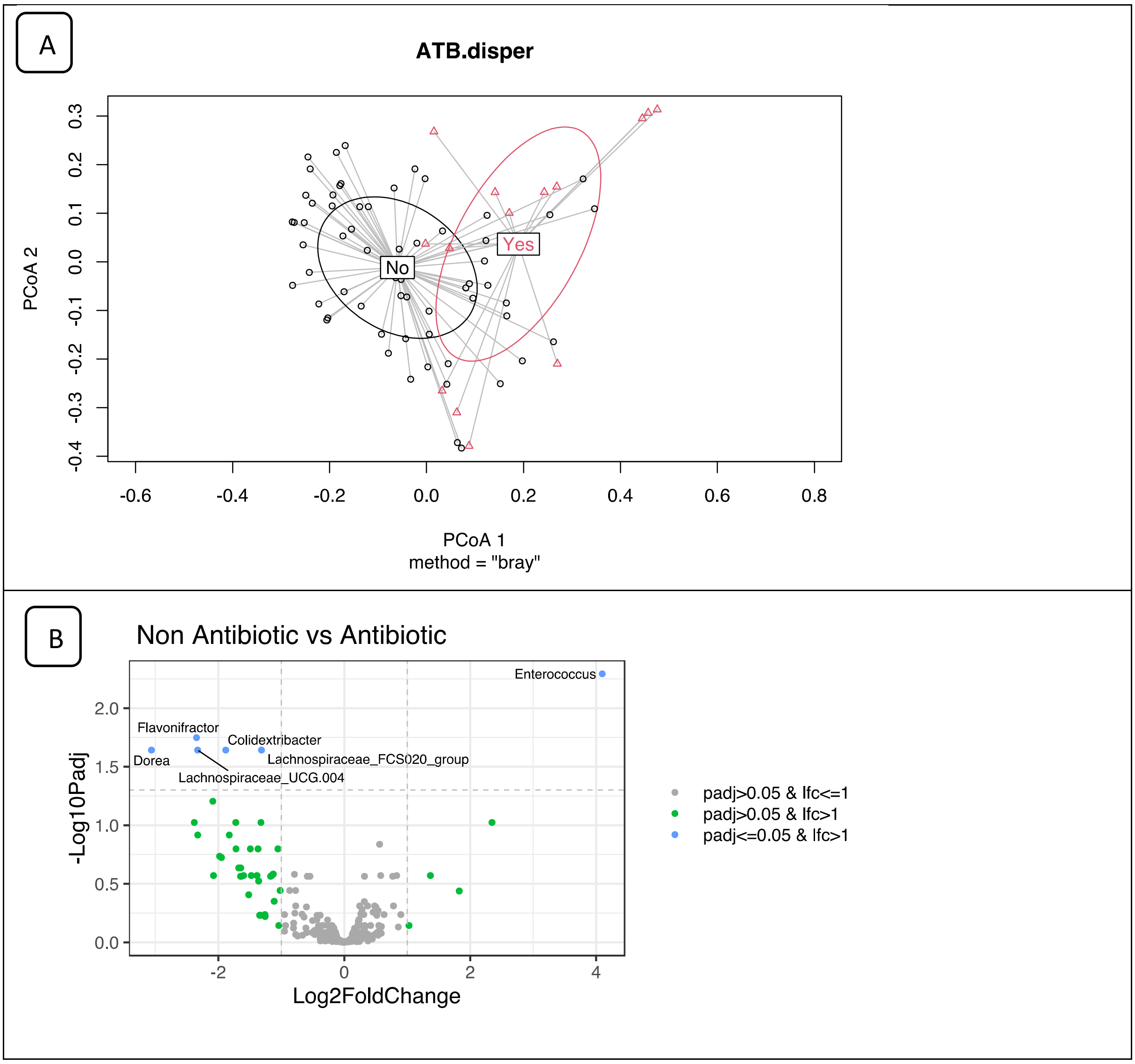

Figure S6 – Antibiotic effect on bacterial microbiota

(A) Analysis of β-diversity using betadisper in Rstudio v4.2.2. Betadisper analyzed beta-dispersion on the basis of Bray-Curtis dissimilarity matrix. Dissimilarity (beta dispersion) between groups was assessed using the $Adonis_2$ test. No-antibiotic used group is represented in black, Antibiotic group is represented in red (*P value* = 0.01331)

(B) Linear model for differential abundance in Rstudio v4.2. (linDA) using antibiotic used and patients as variable. Here a representation of log_2fold change of the antibiotic used group between non antibiotic group. Two thresholds are present: the first one is fixed at $-log_{10}$(padj >0.05) and the second one is fixed at -1 and +1 log_2fold change. The grey dots are the genera with *padj* > 0.05 and with an effect size ≤ 1; the green dots are the genera with *padj* > 0.05 and with an effect size >1 and the blue dots are the genera with *padj* ≤ 0.05 and with and effect size ≤ 1.
